# Use of continuous glucose monitoring in insulin-treated older adults with type 2 diabetes

**DOI:** 10.1186/s13098-023-01225-4

**Published:** 2023-11-23

**Authors:** Silmara A O Leite, Michael P Silva, Ana C R Lavalle, Maria C V Bertogy, Murilo Bastos, Suelen C Vieira Kuklik, Guillermo Umpierrez

**Affiliations:** 1 Cline Research Center, Curitiba, Brazil; 2 Federal University of Rio Grande, Rio Grande, Brazil; 3 Universidade Positivo, Curitiba, Brazil; 4 Emory University, Atlanta, USA

## Abstract

**Background:**

Few studies have reported the adherence to and efficacy of continuous glucose monitoring (CGM) for improving diabetes management in insulin-treated older adults with type 2 diabetes mellitus (T2DM).

**Methods:**

Prospective observational cohort study using FreeStyle Libre Flash CGM in insulin-treated adults > 65 years with T2DM and HbA1c between 7% and 9%. The participants wore the CGM during the 6-weeks study period. The primary outcome was time in range (TIR) between 70 and 180 mg/dL. Secondary outcomes included time below range (TBR), glycemic variability (GV), adherence, and use of glucose data for self-insulin adjustment. Linear regressions with random effects verified the changes in TBR, TIR, time above range (TAR), GV, and GMI across the three visits using CGM (baseline, 4 weeks and 6 weeks), controlled for sex, age, educational level, and health system (private or public).

**Results:**

A total of 66 participants completed the six weeks of CGM (age 72·8 ± 5·3 years; BMI 27·8 ± 3·6 kg/m2), HbA1c: 8·0 ± 0·6%, with an overall sensor utilization of 93·1 ± 6·0%. We observed a stability in TIR (baseline: 63.5 ± 18.9% vs. endpoint: 65.5 ± 18.8%; β = 1,0, p = 0.190). Despite the low TBR at the baseline, we observed statistically significant reduction over the study period (baseline: 5.8 ± 7.0% vs. endpoint: 3.8 ± 4.7%; (β=-1.00, p = 0.008). Glucose variability also reduced from the baseline (34.9 ± 7.2%) to the endpoint (33.0 ± 6.8%) (β=-0.99, p = < 0.001).

**Conclusion:**

FreeStyle Libre Flash CGM is well accepted by older adults with T2DM and allows participants to make therapeutic decisions to reduce TBR and glycemic variability.

## Introduction

The prevalence of diabetes increases with age, reaching 26.8% among subjects aged 65 years and older [1]. The aging of the world’s population has increased the proportion of those with diabetes over the age of 60 years [2]. Given the heterogeneity of older individuals with type 2 diabetes mellitus (T2DM), an individualized approach is warranted to avoid overtreatment and hypoglycemia in frail older individuals and the undertreatment of otherwise healthy [3].

Increased duration of diabetes is associated with reduced ß cell mass, which leads to insulin deficiency and the need for insulin therapy to achieve glycemic control in many older adults with long-standing T2DM [4, 5]. Insulin therapy, however, increases the risk of hypoglycemia, which is associated with adverse clinical outcomes. Clinical guidelines have recommended higher HbA1c targets for older adults than for younger adults to avoid hypoglycemia, based on safety concerns [6, 7].

A recent study using continuous glucose monitoring (CGM) in older individuals (> 69 years) with poor glycemic control (HbA1c > 8.0%) reported a frequency of hypoglycemic episodes (< 50 mg/dL) in about half of insulin-treated T2DM [8]. Hypoglycemic episodes are difficult to diagnose in older adults and are easily missed by intermittent finger stick measurements [9]. Unfortunately, older adults (≥ 65 years) are underrepresented in clinical trials, [10, 11] and there are limited data regarding the benefit and adherence of CGM in older adults with T2DM [12].

This study aimed to evaluate adherence to and efficacy of CGM in improving diabetes management in insulin-treated older adults with T2DM.

## Methods

### Study design and participants

This prospective observational cohort study was registered at ClinicalTrials.gov (NCT04411277). The local institutional review board (IRB) at Universidade Positivo approved the protocol and provided informed consent. Participants were identified by medical records from the Hospital da Cruz Vermelha Brasileira-Filial do Paraná and neuro geriatrician and endocrinology private clinic. Individuals were invited to participate by phone, and interested participants were referred to the Cline Research Center where the study procedures were carried out. Thus, the sample included patients from both public and private health systems.

### Data collection process

At the first clinic visit to the research center, after signing an informed consent form, the participants were evaluated for their educational level and presence of comorbidities, including cardiovascular disease, hypertension, and dyslipidemia. Microvascular complications were assessed by a previous eye examination for the presence of retinopathy, foot examination for neuropathy, and the estimated glomerular filtration rate (eGFR) was calculated using the CKD-EPI formula.

Screening for the presence of sarcopenia was performed using the SARC-F questionnaire, measuring muscle strength with grip strength using the Saehan dynamometer, and the functional test of getting up from the chair without the support of arms and walking for 3 m, turning 180º, then returning to the sitting position. In addition, body composition analysis using InBody 270 multifrequency bioimpedance was used to calculate appendicular skeletal muscle mass.

Inclusion criteria were age > 65 years, a history of T2DM, BMI between 18·5 and 35 kg/m², HbA1c between 7% and 9%, a stable diabetes medication regimen and body weight over the prior three months, and treatment with insulin and with or without oral agent treatment.

We excluded subjects diagnosed with type 1 diabetes, taking glucagon-like peptide 1 receptor agonists, eGFR < 30 ml/min/1.73 m², anemia (hemoglobin < 11 g), chronic liver disease (ALT > 3x upper limits of normal), use of glucocorticoids within three months prior to the study period, history of neoplasia treatment, and inability by the patient or caretaker to commit to the CGM instructions.

Eligible patients had a CGM sensor (FreeStyle Libre Flash CGM system, Abbott Diabetes Care) inserted for the first time in the posterior upper arm and were recommended to undergo CGM as many times as possible during the day, avoiding scan intervals longer than 8 h. Participants were instructed to return to the research center every 2 weeks, where the CGM data were downloaded and glucose reports were generated for review by the primary provider. CGM monitoring was performed for six consecutive weeks. The first two weeks were considered the baseline, the next two weeks were follow-up 1, and the final two weeks were considered the endpoint.

The research team and healthcare professionals were instructed not to change the treatment regimen except for safety reasons, and in this case, the participants were excluded from data analysis.

### Outcomes measures

The primary outcome was time in range (TIR) between 70 and 180 mg/dL, and secondary outcomes were time below range (TBR) (< 70 mg/dL and < 54 mg/dL), time above range (TAR) (> 180 mg/dL, and > 250 mg/dL), glucose variability (GV) (calculated by % coefficient of variation, %CV = standard deviation (SD)/mean glucose x 100%), and adherence to CGM use (the percentage of captured sensor data). TIR, TBR, and TAR were expressed as percentages of the time per day. We also evaluated the prevalence of TIR ≥ 70% of the day, TBR (< 70 mg/dL) > 4% of the day, TBR (< 54 mg/dL) > 1% of the day, TAR (> 180 mg/dL) > 25% of the day, TAR (> 250 mg/dL) > 5% of the day, and the GV > 36% for each timepoint of the study (baseline, follow-up 1, and endpoint).

### Statistical analysis

We used measures of central tendency (mean and median), variability (SD, interquartile range (IQR)), absolute (n), and relative (%) frequencies to describe participant characteristics and glucose profiles. A set of eight linear regressions with random intercept (participants) and slopes (time points) verified the changes in TBR (Overall: <70 mg/dL; TBR 1 (54–70 mg/dL, and TBR 2 (< 54 mg/dL)), TIR, TAR (> 180–250 mg/dL and > 250 mg/dL), GV, and glucose management indicator (GMI) across the three time points (baseline, follow-up 1 and endpoint) controlled by gender, age, educational level, and health system (private or public). We further explored the covariance between the intercept and slope to identify the influence of baseline values on the changes over time. The results were expressed in regression coefficients (β) and 95% confidence intervals (95% CI). The statistical significance was set at p < 0.05. The sample size showed a power of 0.80 to identify regression coefficients equal to or higher than ± 0.43 with an alpha of 95%. All statistical analyses were performed using Stata MP 14.1.

## Results

**Fig. 1:**
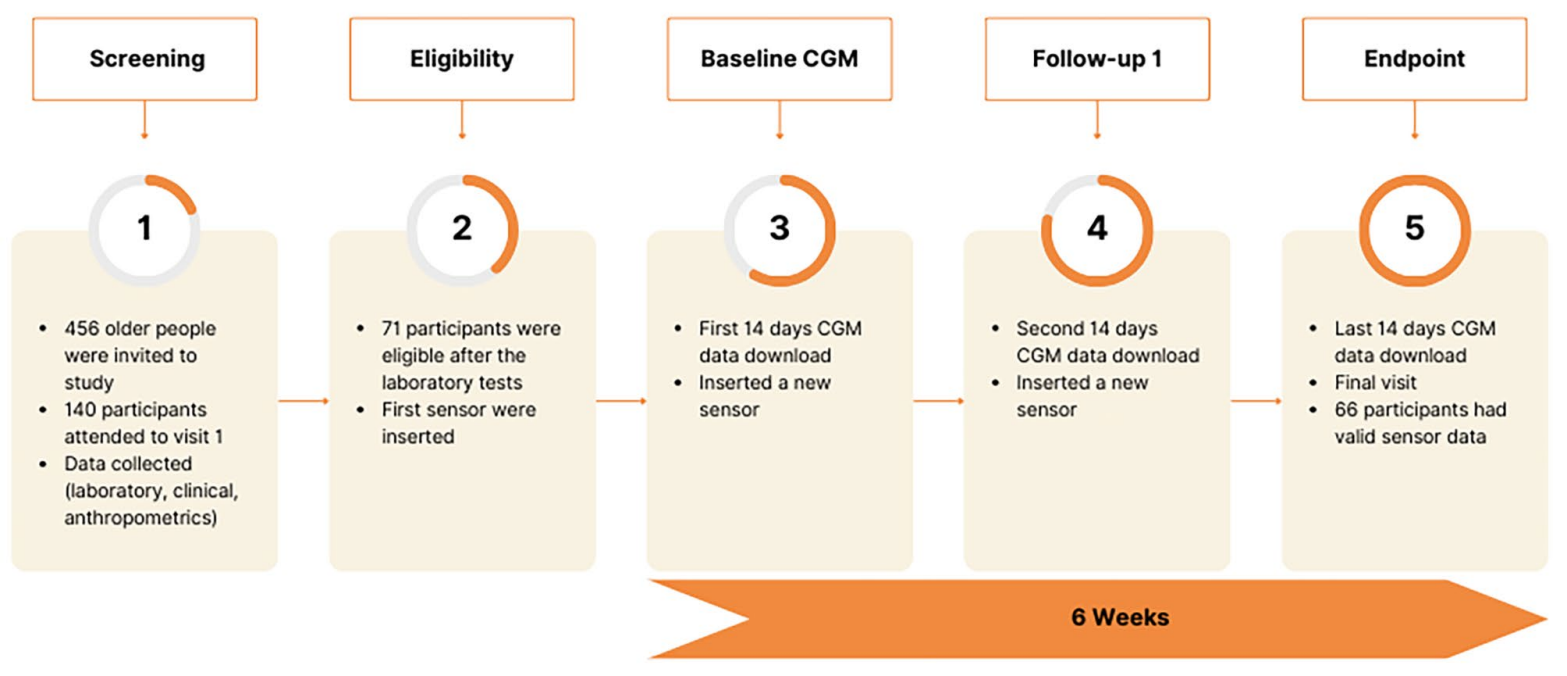
Study design

From August 2020 to April 2022, 456 potential participants were invited to participate in the study by phone. A total of 140 subjects completed their 1st visit to undergo a detailed medical history, physical examination, assessment of diabetes complications, sarcopenia, and laboratory tests. The exclusion criteria eliminated 49.3% of the participants, leaving 71 eligible participants to start the CGM program. Five subjects did not have valid sensor data (< 70% readings) and were excluded from the final analysis. (Fig. 1) Participants who did not have valid sensor data were mainly female with a mean age of 71·4 ± 7·5 years. There were no significant differences in the clinical characteristics between the dropout participants and those included in the final analysis (p > 0·05).

### Baseline clinical characteristics

Table 1 presents the baseline patient characteristics. The sample was equally composed of females and males (50%), with a mean age of 72.5 ± 5.3 years, a mean T2DM duration of 20 ±10.2 years, and a mean HbA1c of 7·9 ± 0·6%. The prevalence of obesity (BMI > 30 kg/m2) was 27.3%. A total of 39.4% reported regular physical exercise, 6.1% were smokers, and 28.9% consumed alcohol regularly. Regarding diabetes complications, 50% had been diagnosed with cardiovascular disease, 33.3% with heart failure, 18.2% with retinopathy, 36.4% with nephropathy, and 56.1% had peripheral neuropathy.

**Table 1:** Patient’s characteristics (n = 66)

	Results
**Gender (female)** (n (%))	33 (50.0%)
**Age (y/o)** (mean ± SD)	72.5 ± 5.3
**Educational Level**	
Elementary school	25 (37.9%)
Middle school	11 (16.7%)
High school	13 (19.7%)
College	17 (25.7%)
**T2DM duration(years)**(mean± SD)	20.0 ± 10.2
**BMI (kg/m²)** (mean ± SD)	27.8 ± 3.6
**Obesity (BMI > 30)** (n (%))	18 (27.3%)
**Body fat (%)** (mean ± SD)	34.1 ± 7.9
**Visceral fat (%)** (mean ± SD)	12.1 ± 4.1
**Sarcopenia** (n (%))	0 (0. %)
**A1C (%)** (mean ± SD)	7.9 ± 0.61
**ALT** (mean ± SD)	21.4 ± 9.9
**AST** (mean ± SD)	21.2 ± 5.0
**Cr (mg/dL)** (mean ± SD)	2.6 ± 12.7
**eGFR** (mean ± SD)	64.3 ± 16.0
**Regular exercise**, n (%)	26 (39.4%)
***Diabetes *** **Complications**	
CVD presence, n (%)	33 (50.0%)
High Risk CVD*, n (%)	40 (60.6%)
Heart Failure, n (%)	22 (33.3%)
Retinopathy, n (%)	12 (18.2%)
Nephropathy (eGFR < 60), n (%)	24 (36.4%)
Neuropathy (n (%), n (%)	37 (56.1%)
**Public Health System**, n (%)	38 (59,1%)

* Hypertension + Dyslipidemia + T2DM

Table 2 shows information on diabetes treatment. Metformin was the most common oral antidiabetic drug (OAD) used by 78.8% of participants, and iSGLT2 was used by 22.7% of patients. Regarding the insulin regimen, 80% of the participants used human NPH (57.6%) or NPH + Regular (21.2%) formulations.

**Table 2:** Diabetes treatment (n = 66)

	n (%)
**Oral Antidiabetic Drugs (OAD)**	
SGLT2i	15 (22.7%)
Sulfonylureas	42 (63.6%)
DPP-4i	11 (16.7%)
TZDs	0 (0.0%)
Metformin	52 (78.8%)
No OAD	8 (12.1%)
***Insulin Regimens + OAD***	82.1%
NPH	38 (57.6%)
NPH + Regular	14 (21,2%)
Degludeca	1 (1.5%)
Degludeca + Rapid Analog	1 (1.5%)
Glargine	5 (7.6%)
Glargine + Rapid Analog	7 (10.6%)
Premix	0 (0,0%)

### Sensor adherence

Overall, the sensor adherence reached 93%. Five (7.0%) participants had the CGM sensor capturing data less than 70% of the time at least in one CGM evaluation (baseline, follow-up 1 or endpoint).

The mean % time of the active sensor was > 91% for all the visits (overall: 92.6% ± 8.3). Sensor issues occurred in only 8% of the total sensors used (n = 213) (Table 3). There were 17 sensor issues; the most common problem was the sensor fall-offs, accidental pull-offs (n = 12, 70.0%). None of the patients experienced the skin symptoms/sensor-insertion events expected with device use (erythema, itching, and rash).

**Table 3:** Sensor adherence and issues

	Baseline	Follow-up 1	Endpoint	Overall
**Sensor readings > = 70%** (n (%))	68 (95.8%)	71 (100.0%)	69 (97.2%)	66 (93.0%)
**Sensor issues** (n (%))	9 (12.7%)	5 (7.3%)	3 (4.3%)	17 (8.0%) ^a^
**Active sensor (%)** (mean ± SD)	91,0 ± 11.3	93.9 ± 5.0	92.9 ± 7.4	92.6 ± 8,3 ^a^

^a^: calculated using the overall number of observations (n = 213, 71 at each visit)

### Glucose profile

**Fig. 2:**
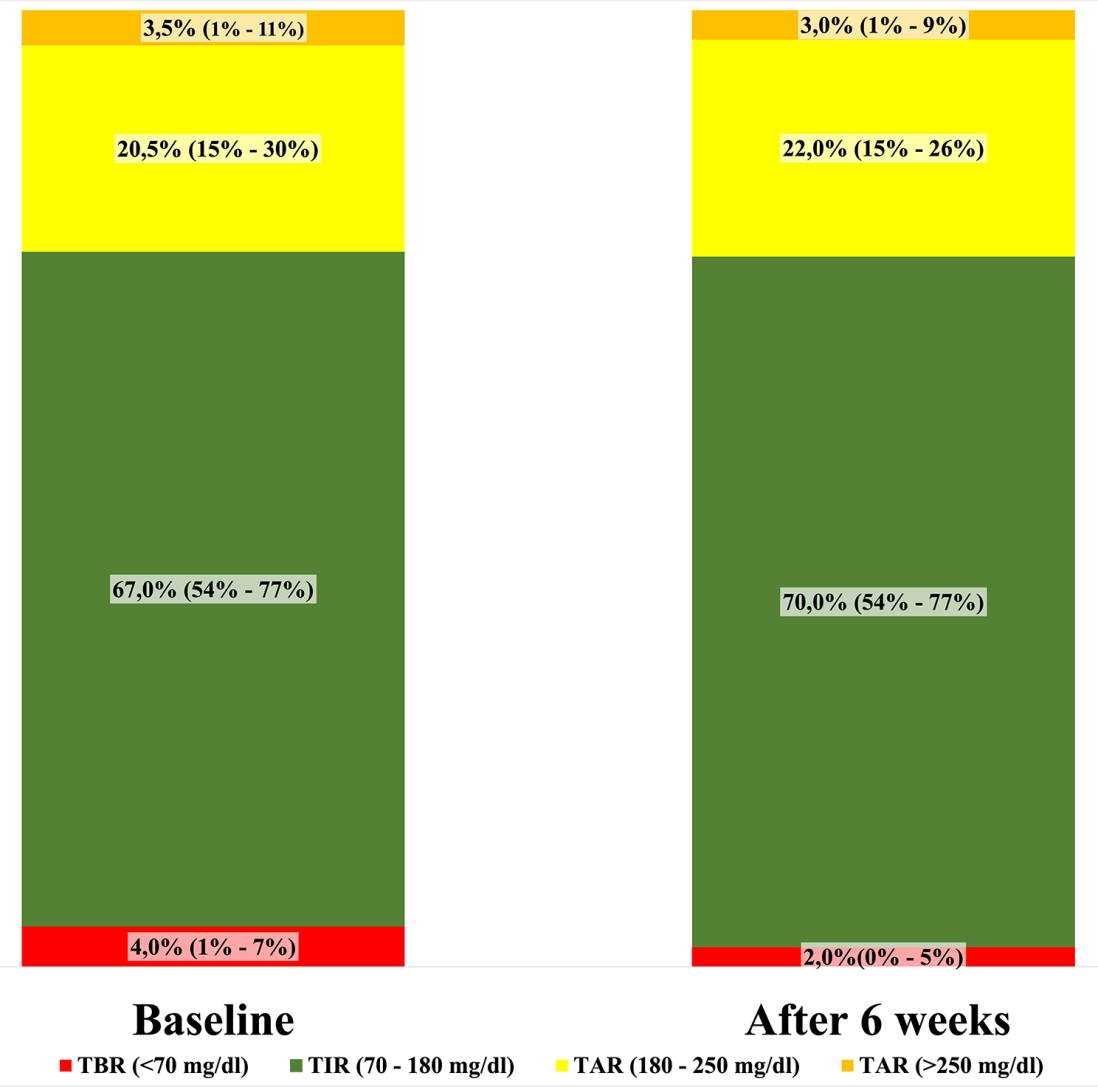
Glucose profile at baseline and after 6 weeks sensor use

Insulin-treated older adults at baseline had a median TIR of 67.0% (IQR = 52.0–77.0), median TBR of 4.9% (IQR = 1.0–7.0), median TAR (180–250 mg/dL) of 20.5% (IQR = 15.0–30.0), and median TAR (> 250 mg/dL) of 3.5% (IQR = 1.0–11.0). The mean glucose variability (GV) was 34.5% ± 7.2 and the mean GMI 7.0 ± 0.8. After six weeks of CGM, the median TIR was 70% (IQR = 57–76), TBR was 2% (IQR = 0–5), TAR (180–250 mg/dL) was 22% (IQR = 15–26), and TAR (> 250 mg/dL) was 3% (IQR = 1–9) (Fig. 2).

**Fig. 3:**
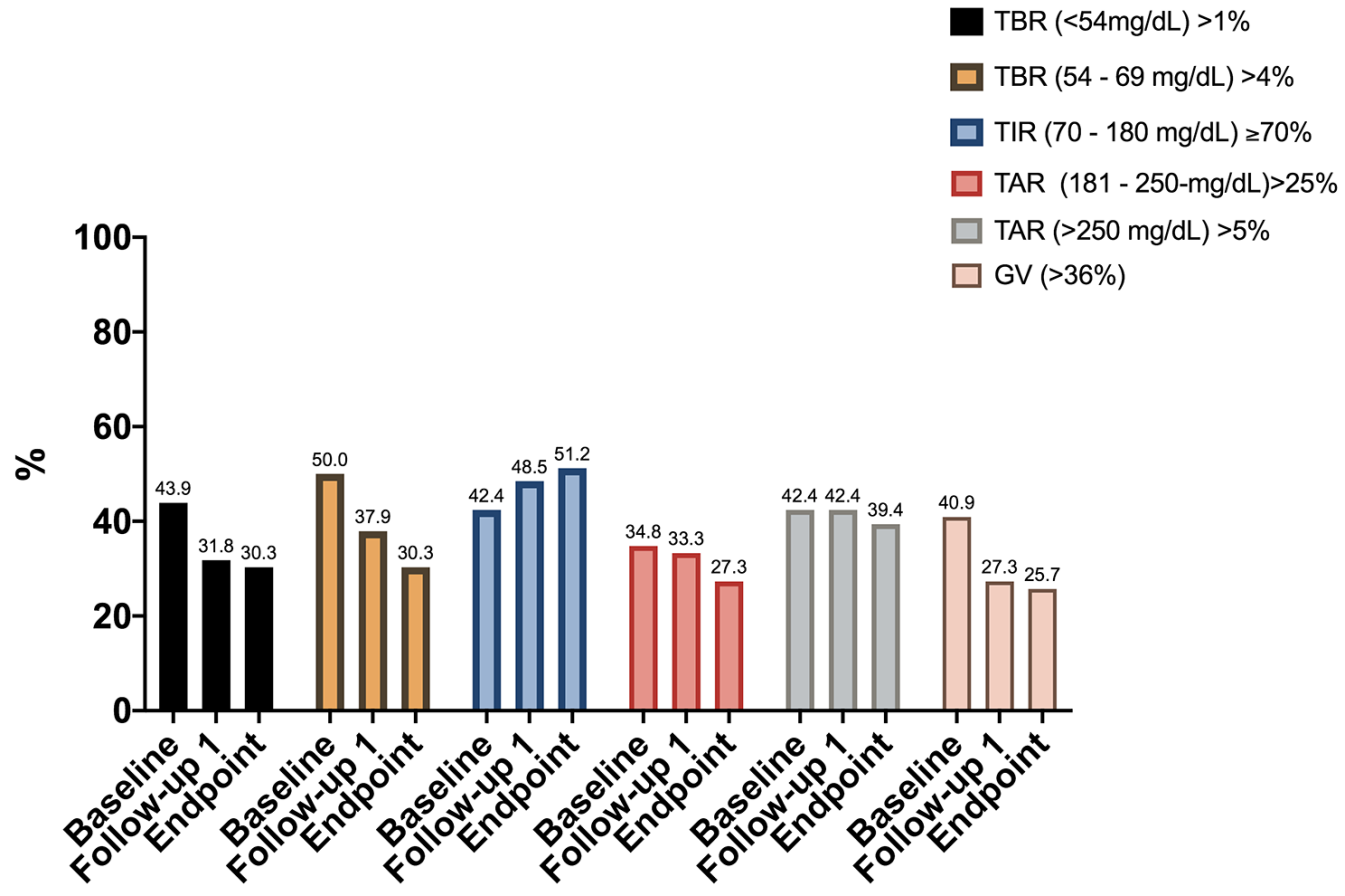
Prevalence of recommended target for TIR and above targets for TBR, TAR and GV from baseline to endpoint

TIR ≥ 70% of the day occurred in 42.4% of the participants at the baseline and 51.5% at the endpoint. TBR (< 54 mg/dL) > 1 of the day occurred in 43.9% of the participants at the baseline and in 30.3% at the endpoint. TBR (54–70 mg/dL) > 4% of the day occurred in 50.0% of the participants at the baseline and in 30.3% at the endpoint. TAR (180–250 mg/dL) > 25% of the day occurred in 34.8% of the participants at the baseline and in 27.3% at the endpoint. TAR (> 250 mg/dL) > 5% of the day occurred in 42.4% of the participants at the baseline and in 39.4% at the endpoint. GV > 36% occurred in 40.9% of the participants at the baseline and in 25.7% at the endpoint (Fig. 3).

Table 4 shows the mean, SD, and regression coefficients for TIR, TBR, TAR, GV, and GMI changes during the study period. Significant changes were observed only in the TBR and GV. TBR (< 70 mg/dL) decrease on average 1% (95% CI= -1.75; -0.26, p = 0.008), TBR 1 (54–70 mg/dL) decreased on average 0.57% (95% CI= -1.01; -0.13, p = 0.012), TBR 2 (< 54 mg/dL) decreased on average 0.43% (95% CI= -0.83; -0.03, p = 0.035). GV decreased by 0.99% (95% CI= -1.53; -0.44, p < 0.001) by visit. There was a negative covariance between the intercept and slope for both variables (TBR and GV), indicating that patients with higher TBR and GV values at baseline showed a greater reduction in these variables.

**Table 4:** Changes in glucose profile during the study period(n = 66)

	Baseline		Endpoint		β*	95%CI	p
Dependent variable	Mean	SD	Mean	SD			
**TBR (< 70 mg/dL) (%)**	5.81	7.05	3.82	4.72	**-1.00**	**-1.75; -0.26**	**0.008**
**TBR 1 (54–70 mg/dL) (%)**	4.06	4.00	2.92	3.44	**-0.57**	**-1.01; -0.13**	**0.012**
**TBR 2 (< 54 mg/dL) (%)**	1.76	3.46	0.89	1.77	**-0.43**	**-0.83; -0.03**	**0.035**
**TIR (70–180 mg/dL) (%)**	63.51	18.92	65.51	18.81	1.00	-0.49; 2.49	0.190
**TAR (> 180–250 mg/dl) (%)**	30.66	20.21	30.66	19.50	-0.05	-0.86; 0.76	0.898
**TAR (> 250 mg/dl) (%)**	8.44	12.33	8.33	12.46	0.05	-0.97; 1.07	0.919
**GV (%)**	34.95	7.20	32.97	6.80	**-0.99**	**-1.53; -0.44**	**< 0.001**
**GMI**	7.00	0.85	7.08	0.81	0.04	-0.01; 0.10	0.133

Baseline: First CGM data; Endpoint: Last CGM data (total: 6 weeks). *Adjusted by sex, age, educational level, and health system

## Discussion

This real-world observational study showed that insulin-treated older adults with T2DM or their caregivers were able to handle Libre Flash CGM regardless of schooling.

We observed stability in TIR from the baseline to the end of the study; despite statistical significance, the prevalence of TIR > 70% increased by 9.1 p.p., which might indicate clinically significant changes at the individual level. These data may bring the debate about the recommendation TIR > 50% as a goal to achieve in elderly patients [13]. Therefore, future interventional studies are required to address this issue.

In this study, hypoglycemic episodes were very low in participants with a mean age over 70 years old, a long duration of T2DM, insulin treatment, and a high prevalence of macrovascular complications since the first 14-day baseline CGM use. These results suggest that CGM use may help patient self-management, improve level 1 hypoglycemia detection, and prevent severe hypoglycemia. Older patients with T2DM are at particularly high risk of developing severe hypoglycemic episodes [9]. Further studies should be conducted using CGM as a tool to prevent hypoglycemic events in elderly insulin-treated patients.

Even without a formal diabetes Flash CGM self-management education program, TBR decreased over the 6 weeks in parallel with glycemic variability improvement from the baseline to the end of the study. The improved safety of insulin treatment in this high-risk group of patients is in line with a previous publication showing the use of flash CGM is associated with reduced diabetes events and hospitalizations in insulin treated T2DM [14]. We assumed that reductions in GV occurred mainly because of decreases in TBR, since TAR did not change over the study period. Skin integrity in the elderly was not an issue for Flash CGM use, and there were no transmission errors from the sensor. Older adults with T2DM whose sensor adherence was greater than 70% were able to make the correct decisions regarding glucose management. There have been few efficacy trials of real-time CGM involving insulin-treated older patients with T2DM [15]. Consequently, there is no official recommendation in the ADA’s *Standards of Medical Care in Diabetes Technology-*2023 for using CGM in insulin-treated older adults with T2DM [16].

This study demonstrates that Flash CGM is a good tool to improve diabetes care in older people, since they were able to make correct therapy decisions, experienced few sensor issues during the 6 weeks of the study, and none of them were related to subcutaneous conditions. This study has some limitations, including the small number of participants. The explanation for this is that the recruitment was done during the COVID-19 pandemic, and the study was conducted in a single center with a short-term follow-up. There were no data showing the glucose profile with blinded sensor wear, and the Freestyle Libre Pro was not available in Brazil at the time of this study. Larger clinical trials with longer durations are required to assess the persistent benefits of this intervention. In addition, the CGM sensor placement was performed at the center; therefore, we did not assess the elderly’s ability to manage it by themselves. Another limitation of the present study was the lack of data on the frequency of viewing instantaneous CGM information by patients. Future investigations should collect this information, because it may be related to better glycemic control.

## Conclusion

The use of Flash CGM in older people with type 2 diabetes receiving insulin treatment is possible regardless of social conditions or schooling, improving the glycemic profile and safety in conditions of high risk of hypoglycemia.

## Data Availability

The datasets used can be accessed from the Cline Research Center’s study source document file.
